# Inorganic A-site cations improve the performance of band-edge carriers in lead halide perovskites

**DOI:** 10.1007/s12200-023-00078-z

**Published:** 2023-09-25

**Authors:** Cheng Wang, Yaoguang Rong, Ti Wang

**Affiliations:** 1 Key Laboratory of Artificial Micro- and Nano-Structures of Ministry of Education, School of Physics and Technology, Wuhan University, Wuhan, 430072 China; 2 Wuhan National Laboratory for Optoelectronics, Huazhong University of Science and Technology, Wuhan, 430074 China

**Keywords:** Perovskite, Inorganic cations, Carrier diffusion

## Abstract

In lead halide perovskites, organic A-site cations are generally introduced to fine-tune the properties. One of the questions under debate is whether organic A-site cations are essential for high-performance solar cells. In this study, we compare the band edge carrier dynamics and diffusion process in MAPbBr_3_ and CsPbBr_3_ single-crystal microplates. By transient absorption microscopy, the band-edge carrier diffusion constants are unraveled. With the replacement of inorganic A-site cations, the diffusion constant in CsPbBr_3_ increases almost 8 times compared to that in MAPbBr_3_. This work reveals that introducing inorganic A-site cations can lead to a much larger diffusion length and improve the performance of band-edge carriers.

**Graphical Abstract:**

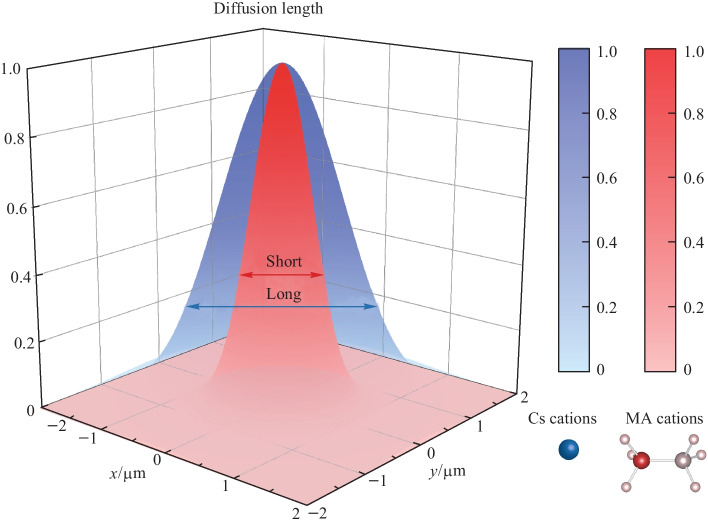

**Supplementary Information:**

The online version contains supplementary material available at 10.1007/s12200-023-00078-z.

## Introduction

With a typical chemical formulation of ABX_3_, lead halide perovskites (LHP) have been demonstrated as promising semiconductors due to their remarkable optoelectronic properties, including long carrier lifetime, long diffusion length, high absorption coefficient, and photoluminescence (PL) efficiencies [1–4]. With these outstanding features, tremendous efforts have been made to fabricate LHP-based devices, such as light-emitting diodes, lasers, and solar cells [5–8]. Notably, the conversion efficiency records for solar cells are continually being refreshed, leaping from 3.8% in 2009 to 25.8% recently [9, 10]. During this development, the A cations of the LHP have been limited to methylammonium (MA^+^), formamidinium (FA^+^), and cesium (Cs^+^) due to the tolerance factor. In conventional thoughts, the A-site cations cannot directly contribute to the LHP band-edge and hardly affect the optoelectronic properties [11–13]. However, some studies have proposed that fast motions of A-site cations are responsible for carrier trapping and electron–hole recombination [14–16]. In addition, polar methylammonium organic cations have been demonstrated to have the ability to detune state coupling and extend hot carrier lifetime [17]. Moreover, fine-tuning of A-site cations is an effective way to improve the structure stability, which is essential for the industrialization of LHP-based devices [18]. For example, inorganic cesium lead perovskite (CsPbX_3_) has better tolerance of humidity, temperature, light, and voltage [19–21]. Although the stability of the materials has been improved with inorganic A-site cations, it is still unclear how inorganic A-site cations impact the carrier transport as well as the device performance in LHP.

Recently, perovskite single-crystal nanostructures have attracted attention owing to their advantages in size and optoelectronic properties [8, 22]. Traditional techniques, such as the Hall effect, time-of-flight, and PL quenching, have limitations in revealing carrier transport properties in an individual nanostructure perovskite [3, 23, 24]. Tian et al. used time-resolved and PL-scanned imaging microscopy to illustrate the carrier diffusion process in nanowire and nanoplate perovskites [25]. Hu et al*.* investigated the electric field-modulated PL imaging method to study the carrier transport in perovskite nanoplates [26]. However, these two techniques need a vigorous PL intensity from samples to achieve a high signal-to-noise ratio. Recently, transient absorption microscopy (TAM) has been demonstrated to be an efficient way to directly visualize the carrier diffusion process, and many studies have been carried out on organic materials, 2D materials, and perovskites [27–29]. To answer the questions whether inorganic cations are essential to the performance of lead halide perovskites, here we investigate the band edge carrier dynamics and diffusion process of MAPbBr_3_ and CsPbBr_3_ single crystal microplates. With the replacement of inorganic Cs^+^ cations, CsPbBr_3_ presents faster bulk recombination dynamics and a larger diffusion constant for the band edge carriers. Due to the high improvement of diffusion constant, the calculated diffusion length of CsPbBr_3_ band edge carrier is much larger than that of MAPbBr_3_. This work highlights that introducing inorganic Cs^+^ cations can benefit the carrier extraction and may achieve excellent photovoltaic performances.

## Experimental

The synthesis of MAPbBr_3_ and CsPbBr_3_ microplates followed our previously reported methods [30, 31]. Specifically, the MAPbBr_3_ microplates were synthesized by immersing a PbAc_2_-coated glass slide in a 7 mg/mL MABr solution in isopropanol at room temperature (22 °C) for about one day, with the PbAc_2_ coated side facing down. The PbAc_2_ thin film was prepared by drop-casting 100 mg/mL PbAc_2_·3H_2_O aqueous solution on a glass slide and dried at 60 °C. The CsPbBr_3_ microplates were synthesized in a home-built chemical vapor deposition system. The ground powders of CsBr and PbBr_2_ (molar ratio 1:1) were mixed and used as precursors for CsPbBr_3_ and placed at the center of the heating zone. Phlogopite mica [KMg_3_(AlSi_3_O_10_)F_2_] was used as a growth substrate and placed downstream of the cooling area. The Argon gas was used as the carrier with a flow rate of 12 sccm and the pressure inside the tube was maintained at 80 mTorr. The center of the heating zone was set to 350 °C and the growth time was ~ 1 h. Note that the growth condition tended to yield more CsPbBr_3_ microwires than CsPbBr_3_ microplates on the substrate. Optical images of MaPbBr_3_ and CsPbBr_3_ microplates are shown in supplementary materials (Fig. S1).

Transient absorption (TA) spectra of perovskite films were measured by a femtosecond pump-probe system with a home-built TA spectrometer. Laser pulses at 1030 nm with 250 fs duration were generated by a 400 kHz amplified Yb:KGW laser system (PHAROS, Light Conversion Ltd.). The probe beam was a white light continuum beam spanning a 450 to 950 nm spectral region, created by focusing 5% of the 1030 nm fundamental output onto a YAG crystal.

A home-built TAM system was used to measure the carrier diffusion process. Briefly, the output of a high-repetition-rate amplifier (Pharos Light Conversion, 400 kHz, 1030 nm) pumped two independent optical parametric amplifiers (TOPAS-Twins, Light Conversion Ltd.). A mechanical translation stage (Thorlabs, DDS600-E) was used to delay the. Both the pump and probe beams were focused onto the samples by an objective (CFI Apo TIRF, Nikon Inc., 60×, NA 1.40). The probe beam was collected by another objective and was detected by an avalanche photodiode (APD; Hamamatsu, C5331-04). A lock-in amplifier was used to identify the change in the probe transmission (Δ*T*) induced by the pump. A pair of Galvanometer mirrors (Thorlabs GVS012) was used to scan the probe beam relative to the pump beam in space to obtain the carrier propagation images.

## Results and discussion

**Fig. 1:**
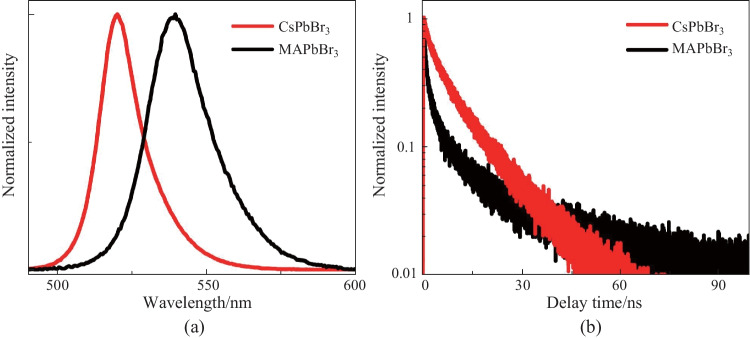
Optical properties of MAPbBr_3_ and CsPbBr_3_ as determined in this study. **a** PL spectra. **b** TRPL. The excitation wavelength is 400 nm

Figure 1a shows the PL spectra of CsPbBr_3_ and MAPbBr_3_. The peak positions of CsPbBr_3_ and MAPbBr_3_ are at 520 and 538 nm, respectively. Although theoretical studies have illustrated that the valence and conduction bands of APbX_3_ are dominated by contributions from the PbX_3_^−^ inorganic sublattice, the A-site cation can fine-tune the lattice parameter and then affect the band gap. As the Cs^+^ cation has a smaller size than the MA^+^ cation, the lattice parameter of CsPbBr_3_ should be smaller than that of MAPbBr_3_. According to band theory, the smaller lattice parameter has a larger band gap, which is consistent with our PL spectra. This subtle difference between the two band gaps has also been demonstrated by experimental and theoretical analysis [32]. Moreover, the full width at half maximum (FWHM) of CsPbBr_3_ is smaller than that of MAPbBr_3_. The broadening of PL in LHP is dominated by trap emission. Smaller FWHM indicates that the trap-assisted nonradiative surface recombination in CsPbBr_3_ is suppressed.

To study the carrier decay dynamics, time-resolved PL (TRPL) kinetics are presented in Fig. 1b. Both the dynamics of MAPbBr_3_ and CsPbBr_3_ fit a bi-exponential decay function. For MAPbBr_3_, a short lifetime of around 1.3 ns and a longer one of around 13.7 ns were observed. CsPbBr_3_ has a shorter lifetime of around 2.4 ns and a longer one of about 11.9 ns. Surface recombination effects have previously been observed in single-crystal perovskite materials [33]. The fast decay is attributed to surface recombination at the surface. However, the surface recombination rate for MAPbBr_3_ was found in the present study to be much faster than that of CsPbBr_3_. This difference may have been caused by the growth method. The CVD growth of CsPbBr_3_ induced fewer defects at the surface. This result is consistent with the PL broadening mentioned above. However, for the MAPbBr_3_, the solution process brought in more surface defects. Excluding the surface recombination process, the slow decay can be attributed to the bulk-free carrier. Previous theoretical work has shown that the electron–hole recombination behavior of MAPbBr_3_ is slower than that of CsPbBr_3_. They conclude that the A-site cation plays a significant role in determining the excited-state lifetime by influencing the nonadiabatic electron–phonon coupling. Thus, the observation here is consistent with the theoretical kinetics.

**Fig. 2:**
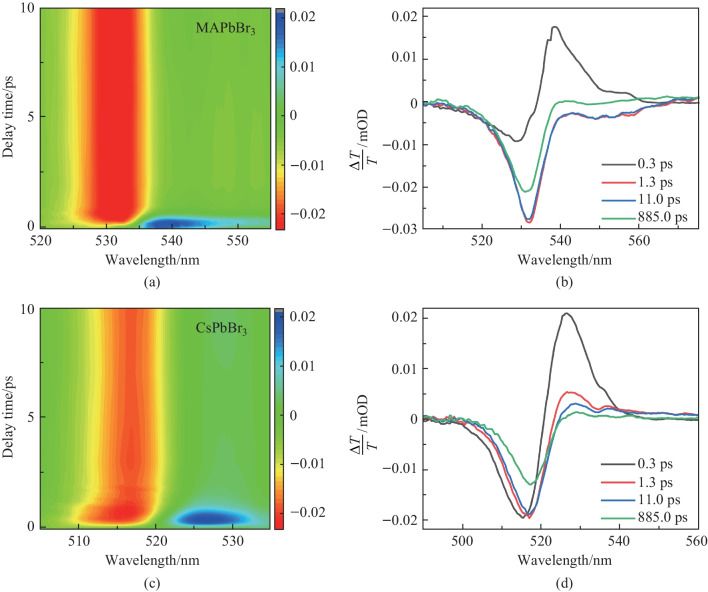
Carrier dynamics of MAPbBr_3_ and CsPbBr_3_. **a** Pseudo TA color image of MAPbBr_3._ **b** TA spectra of MAPbBr_3_ at various delay time. **c** Pseudo TA color image of CsPbBr_3_. **d** TA spectra of CsPbBr_3_ at various delay time. The excitation wavelength is 400 nm

To further study the effects of the A-site cation on the dynamics, TA spectroscopy was performed. Figure 2a, c show the ensemble broadband TA image in pseudo-color plots at early delay time. For both MAPbBr_3_ and CsPbBr_3_, the excitation wavelength was 400 nm. Upon photoexcitation, a ground state bleach band (GSB, the negative signal in Δ*T/T*, and Δ*T* is a pump-induced change in probe transmission, and *T* is the probe transmission) centered around the bandgap at 530 nm was observed due to the band-filling effect for MAPbBr_3_. A photoinduced absorption (PIA, positive signal in Δ*T/T*) band near 537 nm was observed at a delay time shorter than 1 ps (Fig. 2b). Previous works have demonstrated that this PIA peak is related to hot carriers [34]. The TA spectra of CsPbBr_3_ showed similar features to those of MAPbBr_3_, where the GSB and PIA peaks were measured to be at 517 and 526 nm, respectively. The GSB dynamics of MAPbBr_3_ and CsPbBr_3_ were found to be very similar (Fig. S2). For the TA measurements, the transmission mode was applied. The signal was the transmission light after samples, which reflected the features of the bulk sample. However, the reflection mode was used for the TRPL measurements which were more sensitive to the surface. Moreover, since the TRPL dynamics of MAPbBr_3_ was slower than that of CsPbBr_3_, the similar GSB dynamics indicated a faster nonradiative recombination process in MAPbBr_3_.

TAM measurements have previously been demonstrated as an efficient technique to study the carrier diffusion process which can directly visualize the carrier distribution in materials. To analyze the band edge carrier diffusion in these microplates, the pump wavelength was selected at 400 nm for both perovskite materials. The probe wavelengths for MAPbBr_3_ and CsPbBr_3_ were selected for 530 and 517 nm respectively, which were the GSB peaks related to the band edge carrier. Perovskite materials show up to hundreds of ps lifetime of hot carriers due to the hot-phonon bottleneck effect with excitation density higher than 10^18^ cm^−3^ [35–38]. Therefore, a low excitation density was used to eliminate the hot-phonon bottleneck effect, and the diffusion measurement was focused on the transport beyond 2 ps to neglect the hot carrier diffusion effects. Here, all the excitation densities for various pump photon energy were around 1.5 × 10^17^ cm^−3^ which was under the threshold excitation density of the phonon bottleneck effect. Moreover, it is essential to rule out carrier-carrier annihilation effects in transport measurements. If the carrier density at the center of the spot were higher than at the edge, then carrier-carrier annihilation could lead to artificial broadening. We carried out pump fluence dynamics measurements to ensure the impact from annihilation (Fig. S3). It shows similar kinetics with *N*_0_ from 1.5 × 10^17^ to 9.0 × 10^17^ cm^−3^, which suggests that annihilation effects are negligible for the carrier density range here.

**Fig. 3:**
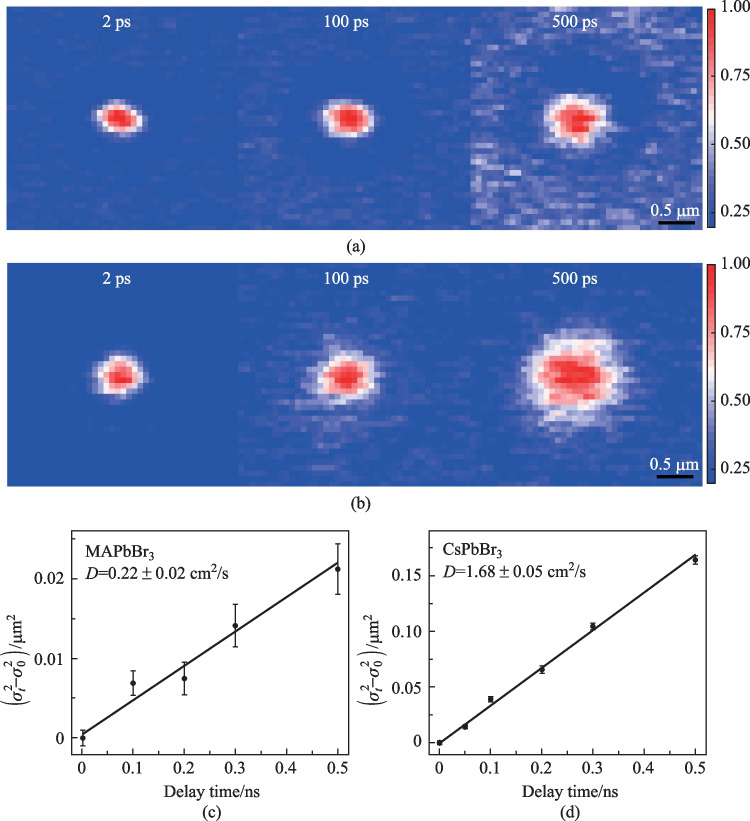
Carrier diffusion of MAPbBr_3_ and CsPbBr_3_. **a** MAPbBr_3_ and **b** CsPbBr_3_ TAM images of the carrier transport at various delay times. The color scale represents the intensity of pump-induced differential transmission (Δ*T*) of the probe and every image has been normalized by peak value. Scale bar: 500 nm. **c** and **d** ${\sigma }_{t}^{2}-{\sigma }_{2ps}^{2}$ plotted as a function of the pump-probe delay time of MAPbBr_3_ and CsPbBr_3_, respectively. Solid lines are the linear fits $D=\frac{{\sigma }_{t2}^{2}-{\sigma }_{t1}^{2}}{2({t}_{2}-{t}_{1})}$

To image the carrier transport process, the pump beam was held at a fixed position while the probe beam was scanned relative to the pump with a Galvanometer scanner and *∆T* was plotted as a function of probe position. The two-dimensional TAM images are shown in Fig. 3a, b for MAPbBr_3_ and CsPbBr_3_ respectively. The initial population was created by a Gaussian pump beam with a pulse duration of ~ 300 fs. At later delay times, the TAM images reflected carrier diffusing away from the initial excitation volume. It is known that the population follows a Gaussian distribution as a function of delay time *t* at low excitation intensity where the high-order recombination terms are negligible. The TAM profiles shown in Fig. 3a, b are fitted by two-dimensional Gaussian functions with variances of ${\sigma }_{t,x}^{2}$ and ${\sigma }_{t,y}^{2}$, where the ${\sigma }_{t,x}^{2}$ and ${\sigma }_{t,y}^{2}$ are the time-dependent variances of the Gaussian profiles along the *x* and *y* axes at delay time *t*. Because the carrier transport is isotropic, we reduce the problem to 1D and define ${\sigma }_{t}^{2}=\frac{{\sigma }_{t,x}^{2}{+\sigma }_{t,y}^{2}}{2}$. The diffusion constant *D* is then given by $D=\frac{{\sigma }_{t2}^{2}-{\sigma }_{t1}^{2}}{2({t}_{2}-{t}_{1})}$. Figure 3c, d plot ${\sigma }_{t}^{2}-{\sigma }_{0}^{2}$ as a function of pump-probe delay time. ${\sigma }_{t}^{2}$ grows linearly as a function of delay time *t* (Fig. 3c, d) as expected for diffusive transport. The carrier diffusion constants of MAPbBr_3_ and CsPbBr_3_ were determined to be 0.22 ± 0.02 and 1.68 ± 0.05 cm^2^/s respectively by fitting the experimental data. This result is consistent with a previous work, which reveals that the carrier diffusion constants of CsPbBr_3_ are 4 times higher than that of MAPbBr_3_ by transient reflection [11].

The diffusion length *L* is an essential parameter for solar cell materials and can be estimated by the diffusion equation $L=\sqrt{Dt}$, where *D* is the diffusion coefficient and *t* is the carrier lifetime. If we calculate the lifetimes from TRPL measurements, the diffusion lengths of MAPbBr_3_ and CsPbBr_3_ are about 0.55 and 1.41 µm, respectively. With the replacement of inorganic cations, the stability of perovskites can be improved. Our results show that the carrier diffusion constant and diffusion length of CsPbBr_3_ also can be boosted compared to MAPbBr_3_. With these fundamental property measurements, we can illustrate that this may be the probable reason for the excellent photovoltaic performances of CsPbBr_3_ solar cells, which have similar performances to those of MAPbBr_3_ solar cells. Large polaron formation has been proposed in hybrid organic–inorganic perovskites, which can effectively screen carrier scattering with optical phonons [39]. For large polaron formation, easy polarization of organic cations with orientational freedom is essential. However, our results show that carriers diffuse faster in all-inorganic CsPbBr_3_ than hybrid organic–inorganic MAPbBr_3_, which indicates that remarkable photophysical and transport properties also exist in all-inorganic perovskites. A previous study on elastomechanical properties of MAPbBr_3_ and CsPbBr_3_ shows that the organic cation makes the entire structure stiffer compared to inorganic perovskite [39]. Therefore, with the replacement by inorganic cations, the lead halide perovskites are still soft and flexible. The soft structure facilitates formation of large polaron which can efficiently screen the carrier scattering with defects and optical phonons regardless of the A-site cation types.

## Conclusions

In conclusion, this work provides insights into understanding the band edge carrier dynamics and diffusion process of MAPbBr_3_ and CsPbBr_3_ single crystal microplates. With the replacement of inorganic cations, both the bulk-free carrier recombination rate and the diffusion constant increase. Besides, the significant property, i.e., diffusion length, is almost 3 times higher than that of MAPbBr_3_. These results suggest that mixing moderate inorganic Cs^+^ cations can enhance the performance of LHP-based devices, not only in structure stability but also in carrier transport. This work reveals an effective way to extend the diffusion length and provides a guide for photovoltaic and other optoelectronics applications of LHP.

### Supplementary Information

Below is the link to the electronic supplementary material.

Supplementary file 1 (PDF 659 KB)

## Data Availability

The data that support the findings of this study are available from the corresponding author, upon reasonable request.
