# Supplementary material for: Inorganic A-site cations improve the performance of band-edge carriers in lead halide perovskites
Source: Front Optoelectron. 2023 Sep 25;16(1):25. doi: 10.1007/s12200-023-00078-z (PMC10519920; doi:10.1007/s12200-023-00078-z)
Supplement: Supplementary file 1 — (PDF 659 KB) [file 12200_2023_78_MOESM1_ESM.pdf]

Supplementary Information

Inorganic A-site cations improve the performance of band-edge carriers in lead halide perovskites

Cheng Wang[1], Yaoguang Rong,[2] Ti Wang[1*]

[1] School of Physics and Technology, and Key Laboratory of Artificial Micro- and Nano-structures of Ministry of Education, Wuhan University, Wuhan 430072, China

[2]Wuhan National Laboratory for Optoelectronics, Huazhong University of Science and Technology, Wuhan 430074, China

* Correspondence: wangti@whu.edu.cn

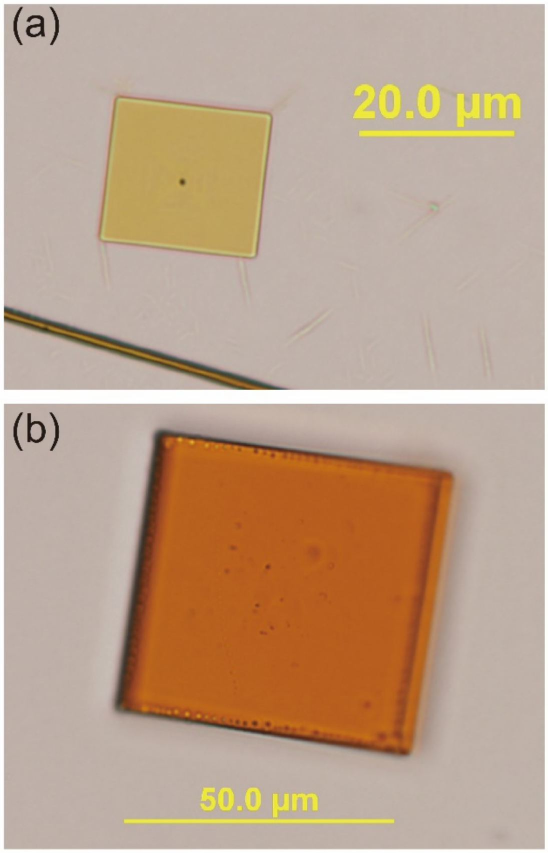

Fig. S1 Optical images of (a) $CsPbBr_3$ and (b) $MAPbBr_3$ microplates.

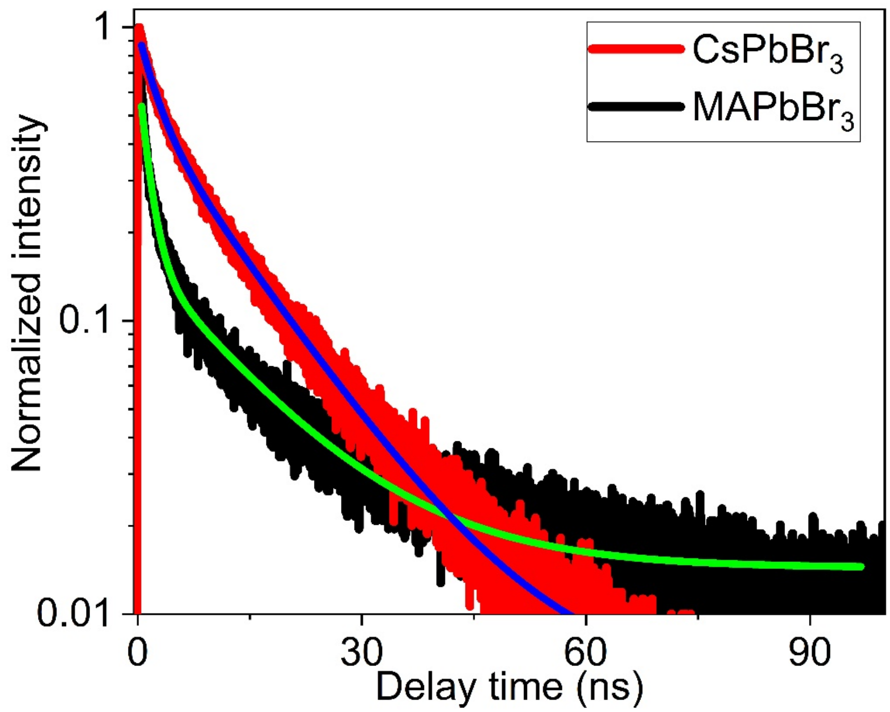

Fig. S2 The blue and green lines are the fitting data of TRPL dynamics of $CsPbBr_3$ and $MAPbBr_3$ respectively. Both of them are fitted by a bi-exponential decay function:

$I(x) = y0 + A_1 \exp(-x/t1) + A_2 \exp(-x/t2)$.

The fitting parameters are presented in the following table:

	$t1$ (ns)	$t2$ (ns)
$MAPbBr_3$	1.34 ± 0.007	13.71 ± 0.078
$CsPbBr_3$	2.40 ± 0.022	11.95 ± 0.047

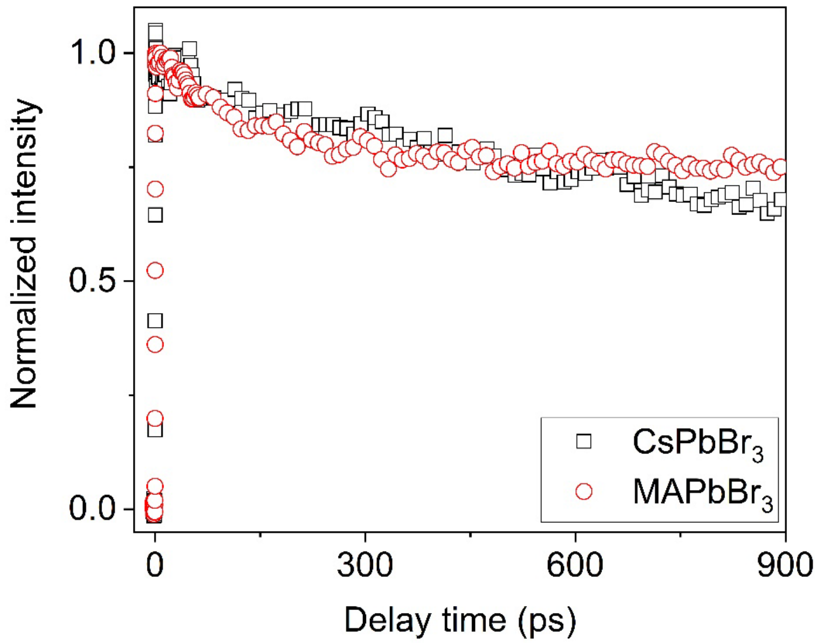

Fig. S3 The dynamics of the GSB peak from TA spectra. The dynamics of $MAPbBr_3$ and $CsPbBr_3$ show similar kinetics.

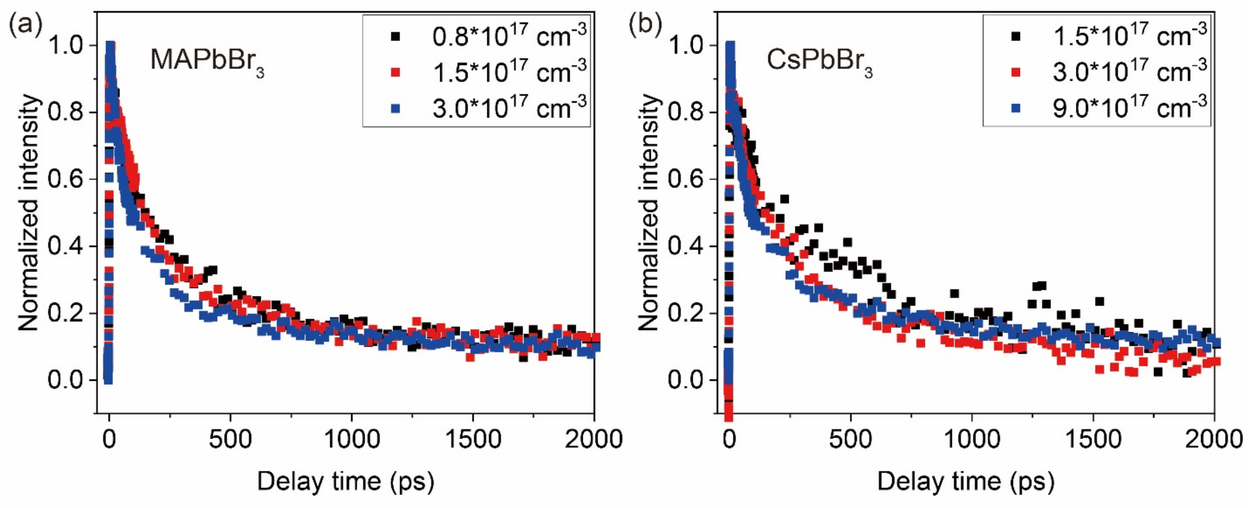

Fig. S4 The normalized TAM dynamics of (a) $MAPbBr_3$ and (b) $CsPbBr_3$ at various pump fluences. Similar kinetics suggest that annihilation effects are negligible here.
